# Implications of Tomophobia and Tomophilia for Clinical Practice: Understanding Patient Attitudes Toward Spine Surgery

**DOI:** 10.7759/cureus.96626

**Published:** 2025-11-11

**Authors:** Bharat R Dave, Mikeson Panthackel, Ajay Krishnan, Shivanand C Mayi, Ravi Ranjan Rai, Mirant B Dave

**Affiliations:** 1 Spine Surgery, Stavya Spine Hospital and Research Institute, Ahmedabad, IND; 2 Spine Surgery, Bhavnagar Institute of Medical Science (BIMS), Bhavnagar, IND

**Keywords:** patient expectations, patient outcomes, supplier induced demand, tomophilia, tomophobia

## Abstract

Advances in spine surgery have dramatically improved safety and precision, yet patient perceptions of surgery now span two psychological extremes: tomophobia, an irrational fear of surgical intervention, and tomophilia, an excessive enthusiasm for operative solutions. This conceptual review explores their historical roots, psychosocial drivers, and clinical implications. Tomophobia leads to treatment avoidance and anxiety, while tomophilia fosters overutilization and surgery beyond indication. Addressing both requires balancing empathy with evidence, emphasizing patient education, and reinforcing ethical decision-making. Recognizing these attitudes can help clinicians navigate the psychological dimension of surgical consent and promote truly patient-centered spine care.

## Editorial

Background

The field of spine surgery has undergone a significant paradigm shift over the past few decades. Advances in imaging, minimally invasive technologies, and precision techniques have revolutionized outcomes and improved safety profiles [1]. However, alongside these clinical gains has emerged an ethical and psychological dilemma: the widening gap between surgical indication and execution. Increasingly, patient perception and physician decision-making are being shaped by psychosocial, cultural, and economic influences extending well beyond evidence-based frameworks [2]. Within this context, the concepts of tomophobia and tomophilia provide valuable psychological and sociological insight into the modern spine patient’s mindset.

Historical perspective and evolution of attitudes

Derived from the Greek *tomo*, meaning “cut” or “section,” the term evokes the essence of surgical practice. During much of the twentieth century, fear of surgery-termed tomophobia-was commonly observed among patients who faced high-risk spinal operations. Limited diagnostic imaging, prolonged recovery periods, and frequent complications reinforced this apprehension. Before the advent of advanced anesthesia and surgical refinement, even routine spinal interventions carried significant fear of paralysis or death. Clinical psychology now identifies tomophobia as an anxiety disorder characterized by avoidance behavior and panic reactions in anticipation of medical or surgical procedures [3]. By contrast, the twenty-first century has ushered in an era of technological confidence. With superior visualization, minimally invasive methods, and enhanced safety, perceptions of surgery have transformed. For some, the pendulum has swung toward overenthusiasm-termed tomophilia-an excessive preference for surgical intervention even when objective indications are lacking. This shift reflects a broader societal embrace of medical technology as a cure-all solution [2,4,5].

Understanding tomophobia and tomophilia

Tomophobia is defined as an irrational, persistent fear of surgery. In spinal practice, this can translate into delayed acceptance or complete refusal of medically warranted procedures. Such patients often present with severe anxiety, psychosomatic symptoms, and a profound lack of trust in clinicians. Management requires multidisciplinary care - psychological counselling, empathic communication, and thorough patient education are essential to restoring confidence [3].

Tomophilia, conversely, is characterized by a disproportionate inclination toward surgical solutions. This attitude is often shaped by social narratives that portray surgery as quick, conclusive, and heroic. Two subtypes are recognized. Type A, or socially influenced tomophilia, occurs when patients demand interventions modelled on friends’ or relatives’ experiences, disregarding individual pathology [6]. Type B, or system-influenced tomophilia, arises when healthcare models, insurance policies, or cost structures unintentionally incentivize surgical overutilization [2,5].

The clinical crisis: surgery beyond indication

Recent literature points toward a concerning rise in spinal surgeries performed without robust evidence-based justification. A prospective analysis reported that nearly 60% of spine patients were offered surgery that was potentially unnecessary [7]. Another study on unnecessary spine surgery noted that about 10% of spinal fusions done under Medicare were not medically indicated [8]. Considering that spinal procedures rank among the most expensive surgical interventions per case, and that the annual economic burden of spine surgery exceeds USD 100 billion, the financial implications of such overuse are substantial [9,10]. Some of the factors contributing to this include patient insistence, medico-legal anxiety, institutional performance pressures, and what some scholars call “supplier-induced demand” - a progressive relaxation of operative thresholds fuelled by technological advancement [11]. The consequences are significant: unnecessary surgery not only exposes patients to risk but also erodes public trust, misallocates resources, and threatens the integrity of surgical discipline.

A case from our own outpatient experience illustrates this growing concern. A patient presented with severe L4-L5 radicular pain but no significant compressive lesion on MRI. Despite the lack of objective indication, the patient underwent surgery at another institution, during which a dural tear occurred, resulting in extended hospitalization, increased financial burden, and avoidable morbidity. This scenario underscores the dangers of overreliance on imaging without clinico-radiological correlation and highlights how deviation from evidence-based decision-making can directly compromise patient welfare.

Implications for spine practice

Addressing these dual extremes demands a multipronged approach integrating clinical rigor with psychological insight. Clinical judgment must remain guided by strict clinico-radiological correlation (CRC). Without precise correlation between imaging and symptoms, even the most technically flawless surgery will yield suboptimal outcomes [12,13]. It is also important to recognize that imaging modalities such as magnetic resonance imaging (MRI) are not always reliable in identifying the true pain generator. Studies have demonstrated that nearly 52% of asymptomatic individuals exhibit abnormal findings on spinal MRI, underscoring the risk for overdiagnosis and overtreatment [14].

Patient education is a cornerstone of surgical ethics. Preoperative counselling should emphasize natural disease progression, conservative modalities, and realistic functional recovery expectations. Some programmes like second opinion programmes and unified referral systems, where the surgeon has no direct access to patients, have been shown to be useful to reduce unnecessary spine surgery [14].

Societal awareness must be strengthened through community education and responsible media engagement to correct misconceptions surrounding surgical “quick fixes”.

Policy reinforcement is essential. Instituting national or institutional oversight, standardized criteria, and peer-review protocols can ensure adherence to evidence-based indications while promoting ethical decision-making [12,13].

Conclusion

Tomophobia and tomophilia symbolize opposite poles of patient response to surgical intervention. Both conditions, though rooted in different psychological mechanisms, can undermine the equilibrium between patient welfare and medical progress. An enduring challenge for modern spine surgeons lies in balancing empathy with evidence and precision with prudence. Ultimately, the goal is not simply to perform surgery well, but to perform it wisely-anchoring surgical excellence in ethical intentionality and patient-centered care.

## References

[1] Galieri G, Orlando V, Altieri R, Barbarisi M, Olivi A, Sabatino G, La Rocca G (2025). Current trends and future directions in lumbar spine surgery: a review of emerging techniques and evolving management paradigms. J Clin Med.

[2] Ellimoottil C, Miller S, Ayanian JZ, Miller DC (2014). Effect of insurance expansion on utilization of inpatient surgery. JAMA Surg.

[3] Schmid M, Wolf RC, Freudenmann RW, Schönfeldt-Lecuona C (2009). Tomophobia, the phobic fear caused by an invasive medical procedure - an emerging anxiety disorder: a case report. J Med Case Rep.

[4] Garfin SR, Fardon DF (2002). Emerging technologies in spine surgery. Spine (Phila Pa 1976).

[5] Berlin NL, Skolarus TA, Kerr EA, Dossett LA (2020). Too much surgery: overcoming barriers to deimplementation of low-value surgery. Ann Surg.

[6] Upp L, Sethi S, Strelzow J, Hynes K (2025). Preoperative expectations for elective vs nonelective foot and ankle patients. Foot Ankle Orthop.

[7] Epstein NE (2013). Are recommended spine operations either unnecessary or too complex? Evidence from second opinions. Surg Neurol Int.

[8] Silverberg LI (2000). Survey of medical ethics in US medical schools: a descriptive study. J Am Osteopath Assoc.

[9] McGirt MJ, Resnick D, Edwards N, Angevine P, Mroz T, Fehlings M (2014). Background to understanding value-based surgical spine care. Spine (Phila Pa 1976).

[10] Ausman JI (2014). The death of spine surgery, sequel - 2014. Surg Neurol Int.

[11] Seyedin H, Afshari M, Isfahani P, Hasanzadeh E, Radinmanesh M, Bahador RC (2021). The main factors of supplier-induced demand in health care: a qualitative study. J Educ Health Promot.

[12] Suh SP, Jo YH, Jeong HW, Choi WR, Kang CN (2017). Outcomes of revision surgery following instrumented posterolateral fusion in degenerative lumbar spinal stenosis: a comparative analysis between pseudarthrosis and adjacent segment disease. Asian Spine J.

[13] Montenegro TS, Gonzalez GA, Saiegh FA (2021). Clinical outcomes in revision lumbar spine fusions: an observational cohort study. J Neurosurg Spine.

[14] AlAli KF (2023). Unnecessary spine surgery: can we solve this ongoing conundrum?. Front Surg.

